# Retraction: The integration of the neurosciences, child public health, and education practice: hemisphere-specific remediation strategies as a discipline partnered rehabilitation tool in ADD/ADHD

**DOI:** 10.3389/fpubh.2025.1633415

**Published:** 2025-06-04

**Authors:** Frontiers Editorial Office

The journal retracts the article cited above.

Following concerns regarding the originality of the article, an investigation was conducted in accordance with Frontiers' policies. It was found that the complaint was valid and that the article should be retracted, because of an unacceptable level of similarity to the article “The effect of hemisphere specific remediation strategies on the academic performance outcome of children with ADD/ADHD” published in International Journal of Adolescent Medicine and Health.

Following the Committee on Publication Ethics guidelines, this article, published most recently, is retracted.

This retraction was approved by the Chief Editors of Frontiers in Public Health and the Chief Executive Editor of Frontiers. The authors received a communication regarding the retraction and had a chance to respond. This communication has been recorded by the publisher.

